# Progress in Surgery

**Published:** 1896-10-17

**Authors:** 


### Progress in Surgery.

### GENERAL SURGERY.

**Fractures and Dislocations**.—The clinical and surgical aspects of the new rays, discovered by Professor Röntgen, of Würzburg, have no better use than in the elucidation of fractures in the living body. The methods of application have lately become more perfect, so that fractures and dislocations may be differentiated even in the thicker parts of the body. It has also been found to be of much use in obscure cases with great swelling, for the swelling is not impermeable to the rays. As W. W. Keen[18] points out, suspected greenstick fractures can be made evident, and in any case in which possibly a claim for malpractice would be made, if, after the bones are adjusted, they were skiagraphed through the dressings, including even the ordinary board splints (plaster, of course, would not do), such a skiagraph might be of value in a court of law. Even if not needed for such medico-legal purposes, it would be of value to the surgeon in assuring him that everything is in proper order. A *résumé* of some of the surgical aspects of the new photography up to date is given by A. Carless in the March number of the *Practitioner*,[19] while Mr. Sydney Rowland[20] continues up to the present time his series of now well known and historic articles upon the whole subject. He says, it is almost impossible, considering the early and undeveloped stage which the new process must be considered to have reached, to predict what will be the ultimate outcome of this invention. It is not too much to say, however, that it will probably, to a large extent, in the near future, replace all other methods in the diagnosis of bone diseases. It will probably settle many vexed questions in surgical pathology and diagnosis. To the same effect, Howard Marsh[21] remarks that in cases of injury to the long bones, and especially of injuries involving the articular ends of the bones, and when it is a question what the precise injury which has been suspected may be, the method will often be valuable in the highest degree. Reclus's method of "systematic conservatism" in the treatment of severe injuries of the limbs, described in a previous number of The Hospital, is illustrated by seven cases in the "Revue de Chirurgie."[22] The author holds that the admirable results which were obtained would not have been possible had the ordinary treatment by amputation been followed. Shock is greatly diminished, while there was no instance of diffuse suppuration, purulent infection, or septicæmia. Death from tetanus, however, occurred in one. Reclus maintains that amputations for traumatism should be done away with.

*Upper Extremity.*—Professor Struthers,[23] in concluding his article on separate acromion process simulating fracture, is of opinion that the occasional occurrence of the injury being symmetrical does not destroy the fracture theory. Seeing that both shoulders may be dislocated simultaneously, there need be little difficulty in believing in the occurrence of symmetrical fracture of the acromion process. The epiphysis theory is attractive to the anatomical mind, but when the evidence is critically examined has to be abandoned for the fracture theory. The explanation given of the frequency of the locality in which the fracture is found to have occurred —the post-clavicular line—giving the family likeness to the great majority of specimens, is, however, no less an anatomical one, and appears to Dr. Struthers to be the true one. A case of fracture dislocation of shoulder, reduced by McBurney's hook-traction method, is reported by T. S. K. Morton.[24] A man, aged seventy-four, fell from a trolley-car upon his shoulder; a dislocation of the head of the humerus being diagnosed reduction was attempted, but almost at once a snap was heard, and evidences of fracture of the humeral neck presented. An operation was, therefore, undertaken through the deltoid muscle on the outer aspect of the joint. A comminuted fracture of the shaft was found to exist, with a long spicule still connected with the inner side of the head. Owing to fatty degeneration the head could only, after two attempts with the traction hook and finger pressure, be dislodged from beneath the clavicle, and replaced in the glenoid cavity. Silver wire sutures were employed to hold the head and shaft in proper position. The patient did well until the eighth day, when he died with sudden collapse, after getting out of bed to sit in a chair. An examination of the wound after death found it to be in perfectly satisfactory condition, with no evidence whatever of infection or other complication. McBurney[25] relates a case of dislocation of the humerus complicated by fracture of the anatomical neck in which considerable time had elapsed since the injury had been received, and probably no non-operative method of treatment would have succeeded in bringing the bone into place. Although he believes that in no case should a major operation be made use of for reducing dislocation which could be reduced by any manipulation of justifiable amount, yet an operation such as he has described was infinitely less grave than the great amount of manipulation which had again and again been practised, and still was frequently practised in reducing dislocations. Two cases illustrating the disabilities resulting from injuries to nerves at the time of fracture, and their subsequent irritation due to displaced fragments, are reported by Dr. G. G. Davis.[26] He is convinced that impairment of function from callus is much less frequent than generally supposed. His experience has been that it is far more commonly due to direct injury of, or pressure upon the nerves by a displaced fragment. The first instance occurred in a boy aged five years, who had fractured his humerus at the elbow six weeks previously. Evidences of fracture could be found in a bony prominence just above the joint, extending from the tendon of the biceps inwards. The contraction of the flexors of the fingers pointed to an implication of the median nerve, and that of the flexor carpi ulnaris showed that the ulnar also was somewhat involved. The median nerve was exposed and found running beside a fragment of bone about three-quarters of an inch long and half an inch broad, firmly fixed to the anterior surface of the humerus. This fragment was drilled away by an electrical drill and the median nerve allowed to resume its normal position, from which it had been pushed by the fragment. At the time of the report, some weeks after the operation, the cure was not yet complete, although the condition was very much improved. In the second case, of a man aged twenty-three years, pain suddenly began at the seat of a fracture of the clavicle and gradually passed down the arm; finally the pain was only felt from the elbow to the fingers. Eight weeks after the accident motion was present only to a very slight degree in the fingers, and the whole extremity was stiffened and the skin glossy. The clavicle was fractured at the junction of its outer and middle thirds, the inner fragment being drawn upwards, and the outer downwards and inwards. An incision was made parallel to the clavicle, and the ends exposed. The fibrous union was cut away and the ends freshened, adjusted, and bound together with silver wire. The wire was removed in seven weeks, and before the eighth week he was discharged, with no pain and good motion in the hand and arm. A backward dislocation of the elbow-joint was most successfully diagnosed by means of the Röntgen photography in a case reported by Mr. Howard Marsh.[27] A subaltern, aged twenty-two, was riding, and his horse fell and rolled over him. Besides the severe concussion of the brain the arm was evidently severely injured in the neighbourhood of the elbow-joint, but there was so much swelling and blood extravasation that it was not possible to venture a precise opinion as to what had occurred. It seemed probable that there was a fracture of the lower end of the humerus extending into the elbow-joint. Mr. Thos. Moore took a skiagraph, which was at once developed, and very completely demonstrated an uncomplicated dislocation. The patient was put under chloroform and the dislocation reduced. Another skiagraph was then taken, and it showed that the displacement had been completely corrected. Mr. Marsh figures both representations of the injury before and after reduction. Another skiagraph taken by Mr. Sydney Rowland[28] seven days after injury represents a rare form of dislocation of the elbow, viz., of both bones outwards. The case was that of a young man under the care of Dr. Edward Gray. No time was lost in reducing the dislocation under chloroform. C. A. Powers[29] discusses the vexed question of position in the treatment of fractures of the lower end of the humerus, and adds the testimony of six hundred and fifty cases under the care of Drs. Hartley and Woodbury, Curtis, Van Arsdale, and the author. He endeavours to demonstrate that the position of safety is that of flexion, and that this position is attended with sufficiently satisfactory results to warrant its being retained by the average practitioner. He thinks, however, that the surgeon who sees many of these fractures will obtain satisfactory results under any form of treatment which his experience and judgment may lead him to adopt, but for the general practitioner, who sees these cases but seldom, to treat them in extension seems to him unwise. Dr. Powers reports five cases treated in the extended position which resulted in anchylosis and loss of function, and necessitated operation.

[18] Am. J. Med. Sciences, Mar., 1896, p. 256. [19] Practitioner, Mar.. 1896, p. 235. [20] B. M. J., Feb. to Aug., 1896. [21] B. M. J., May 16, 1896. [22] Revue de Chirurg, Jan., 1896. [23] Edinburgh Med. Jour., April, June, and Aug., 1896. [24] Annals of Surg., June, 1896. [25] Annals of Surg., April, 1896. [26] Annals of Surg., Feb , 1896. [27] B. M. J., May 30, 1896, p. 1,318. [28] B. M. J., Mar. 7, 1896, p. 620. [29] N.Y. Med. Record, May 2, 1896, p. 615.

### GENITO-URINARY SURGERY.

*(Continued from page 28.)*

**Vesical Calculus.**—Mr. Milton, of Cairo, has given us his experience of lithotrity in a very valuable paper[8], based on the records of 200 of his own cases. Bigelow's method of complete evacuation at one sitting under an anæsthetic had always been employed. An instrument has been invented by the author, and is figured in his paper, for both crushing and evacuating, thus avoiding the change from the lithotrite to catheter; but the lithotrite thus constructed is weakened as a crushing instrument. Its chief use would appear to be in very old men, and patients with enlarged prostates to avoid much manipulation, and the inventor does not use it in ordinary cases of stone. When Milton began to operate for stone he followed the usual custom, and performed supra-pubic cystotomy for stones of unusual size. Out of eleven, five died. But since 1892 all large stones (sixteen cases) have been treated by Bigelow's method, and all recovered. The author says "that the more experience he has of this operation the more he is convinced that there are very few cases indeed which it cannot cope with." He crushed what he believes to be the largest stone ever removed in this way. It was four and three-quarter inches in diameter, and required a specially-constructed lithotrite to grasp it. Its weight was twelve ounces. But for surgeons who only occasionally have to operate for vesical calculus he recommends perineal lithotrity for unusually large stones, an operation which he has performed twenty-seven times with only one death—and performed himself, rather than urethral lithotrity, in order that the students attending the hospital might become acquainted with its details. Of the two methods of performing the operation (*a*) of introducing the lithotrite through an opening into the urethra in the perineum, and (*b*) crushing through an opening into the bladder made as for perineal lithotomy, the author prefers the latter, using a straight, strong pair of forceps as a crusher. He considers that the wound into the bladder should be only of such size as to admit the forefinger, and that no fragments should be removed by the crusher, but small forceps should be employed for the purpose, or the fragments washed out. If they are completely removed, he thinks no fear need be entertained of the persistence of a perineal fistula. Reginald Harrison, who first introduced perineal lithotrity to the profession in 1888, crushes through a perineal incision into the urethra, and has operated on fifteen cases without a death; but Milton believes that the operation cannot be carried out in this way without some laceration of the neck of the bladder.

Milton has met with four cases of stone, partly in the bladder and partly in the ureter, and he thinks that in some cases in which stones have been supposed to have been removed from a bladder sacculus, they have really been of this nature. There is great difficulty in grasping the stone with the lithotrite, and the position of the stone is determined by rectal examination with the sound pressing on the stone in the bladder. In one case the stone was removed by lateral lithotomy, the orifice of the ureter, which tightly embraced the stone, having to be divided; in two others Milton was successful in dragging the stone out of the ureter with the lithotrite, without opening the bladder, and then crushing it; and in the third he dislodged the calculus after opening the bladder above the pubes. He refers to the records of several other cases of stone impacted in the ureteral orifice, which show that the condition is one which may every now and then occur, and he advises that an attempt should be made to drag the stone out of the ureter by the lithotrite, but if this fails he considers that supra-pubic cystotomy should be performed, and the ureteral orifice notched or dilated. The method by which the author believes a stone becomes encysted in the bladder is described. He thinks it is due to localised muscular contraction of the bladder wall where a stone rests, forming a ring-like constriction round it, and he points out, in support of this view that nearly all encysted stones fill the cyst they occupy. He thus describes the process, "For some reason, perhaps during sleep, when the stone probably rests in one place, the muscular contractions become such as to grasp the stone. This contraction may be spasmodic or continuous. In the latter case, after a certain time, the continuous contraction will gradually tend not only to grasp but to envelop the stone. Those portions of the bladder walls which lie immediately beyond the stone, finding no resistance except their own rigidity, will gradually form an annular constriction dividing the bladder into two cavities, one of which is occupied by the stone." He then describes how the orifice of the sacculus may contract, and states that in performing lithotrity he has frequently found the stone just grasped by the bladder wall in some position which it could not occupy as the result of gravity. Milton does not share the opinion of many surgeons that enlargement of the prostate is a contra-indication to lithotrity, for he considers that a post-prostatic pouch when largely open is rather a help than a hindrance to the detection of the stone and the performance of lithotrity. Its tendency to retain crushed fragments may be overcome by reversing a curved evacuator beak into the sac. But in cases where the pouch is more or less separated from the general bladder cavity, by constriction of its upper margin, or an overhanging tumour of the prostate hides the calculus, lithotrity may be impossible, but should, the author thinks, be attempted; and if the stone can be crushed, he would leave the question of dealing with the prostatic growth until after its removal, rather than perform suprapubic cystotomy for the removal of both the stone and prostatic overgrowth, as the latter might be removed by subsequent castration. With regard to lithotrity in children, the author believes everything will depend on the opportunities for operating which the surgeon has. If they are few, he thinks, he had better do lateral lithotomy, as he will not have the necessary ability to perform lithotrity in children, which practice alone can give. In the aged, Milton considers, lithotrity is attended with very much less mortality than any cutting operation, but that the mortality is distinctly high. He thinks that cystitis is not a contra-indication to lithotrity, for his experience has been that the cure of cystitis after lithotrity has been as certain as after lithotomy. As to whether lithotrity or lithotomy should be performed in cases complicated with renal disease, he thinks there is hardly sufficient evidence to show which operation is the best, but he is inclined to give the former the preference.

**Ulceration of the Bladder.**—Henry Fenwick[8] calls attention to the existence of what he calls "the simple solitary ulcer of the bladder." It has been revealed by the use of the cystoscope, and this instrument seems necessary to make the diagnosis with any precision; but the symptoms which Fenwick considers suggestive of its presence are repeated causeless sharp attacks of hæmaturia, with increased frequency of micturition, but only during the daytime, with constant penile pain, without venereal history or evidence of stone or tubercle, and with urine clear and good-coloured, of specific gravity of 1,020. Maternal phthisis, extreme vesical irritability at night, and a pale, neutral, murky urine of 1,010 specific gravity he considers point to tuberculosis. When the bladder is examined with the cystoscope, if the simple solitary ulcer is present, the rest of the bladder is seen to be quite healthy; whereas, if the ulceration is tuberculous, in the posterior wall are seen patches of vivid red, with white flakes of necrotic tissue peeling from them. In tuberculosis the capacity of the bladder is materially reduced, which is not the case with the simple ulcer. These simple ulcers are, Fenwick believes, generally diagnosed as tuberculous, since all ulceration of the bladder is often supposed to be of this nature, and he thinks some of the cases which have been satisfactorily treated by cystotomy and scraping of the ulcerated area have been examples of simple ulceration. A marked feature of the simple ulcer is constant pain at one part of the penile urethra, in some patients increased by exercise, or jolting, or over-holding their water, and a few walk about guarding their penis with their hands to prevent its being shaken or jarred.

The ulcer is found chiefly in young men, and usually close to the orifice of one or other ureter, and occasionally a contact ulcer forms on the anterior part of the bladder just above the urethral orifice, and must be carefully looked out for. Phosphatic deposit not infrequently covers the surface of the ulcer. They last sometimes for years, and when once coated over with phosphates, many resist various local applications, so that cystotomy and scraping is generally required. In the female the curretting can be carried out through a urethral speculum and with the aid of electric light, and may be repeated several times. Occasionally they heal spontaneously. Before phosphatic deposit forms on the surface of the ulcer, there is much more hope of doing good without operation. Fenwick advises the use of sandal oil, followed by maltine and cod liver oil, small doses of mercury at night, liberal diet, and fresh air. Several illustrative cases are recorded.

**Surgery of the Ureter.**—Dr. Willy Meyer, of New York, has given his experiences of catheterism of the ureters in the male and female to the New York Academy of Medicine.[10] The instrument used is a cystoscope, from which a very fine catheter can be projected into the orifice of ureter. Meyer has catheterised the ureters in three female and five male patients, and he is nearly always successful in his search for the ureteral orifices, though they may be drawn aside by intravesical pathological changes or covered by hypertrophied rugæ. The passage of the catheter along the ureter does not cause more pain than urethral catheterism, and the pelvis of the kidney can be safely irrigated, though fluid injected into the lower part of the ureter is apt to set up colicky pain. The author, however, prefers Kelly's method of catheterising the ureters in the female ("Progress in Surgery," The Hospital, November 16th, 1895, page 119), because all the instruments can be boiled. In a contribution to the "Surgery of the Ureter," Bland Sutton[11] calls attention to the fact that rhythmical waves of peristaltic contraction occur in the ureter, the waves passing invariably from the kidney to the bladder. He says it is very fallacious to regard the ureter as a passive conduit, for urine is conveyed along it in the same way that food travels along the œsophagus to the stomach, *i.e.*, by rhythmic contraction. The flow of urine in jets is easily seen from the ureteral orifices in an extroverted bladder. A case in which the bladder became excessively irritable from ureteral obstruction is recorded, which the author explains by the supposition that when the contraction of the ureter becomes exaggerated the contraction is not limited to the ureter, but involves the bladder and thus produces the vesical irritability. A case of hydronephrosis from "inadequate" ureter, in which there was a dragging pain in the left loin with frequent attacks of acute pain resembling renal colic, is also recorded; and the post-mortem dissection of an infant is described, in which the lower end of one ureter was found distended to a much larger size than the bladder, the urethra being impervious. This condition, Bland Sutton thinks, is sometimes wrongly described as sacculation of the bladder.

[8] Brit. Med. Journal, May 9th, 1896, p. 1,133. [9] Lancet, April 18th, p. 1,051; April 25th, p. 1,126; and May 2nd, p. 1,213. [10] New York Med. Record, March 21st, 1896. [11] Lancet, May 9th, 189[illegible], p. 1,275.

